# An African perspective to biodiversity conservation in the twenty-first century

**DOI:** 10.1098/rstb.2023.0443

**Published:** 2025-01-09

**Authors:** Bezeng S. Bezeng, Gabriel Ameka, Chia Michelle Valérie Angui, Laura Atuah, Fortuné Azihou, Yanis Bouchenak-Khelladi, Frank Carlisle, Bi Tra Serges Doubi, Orou G. Gaoue, Wenceslas Gatarabirwa, Consolata Gitau, Craig Hilton-Taylor, Alex Hipkiss, Rodrigue Idohou, Beth A. Kaplin, Lucy Kemp, Jacqueline S. Mbawine, Vincent Logah, Paul Matiku, Paul Kariuki Ndang’ang’a, Eric D. Nana, Onella N. N. Mundi, Erasmus H. Owusu, Jon Paul Rodríguez, Hanneline Smit-Robinson, Kowiyou Yessoufou, Vincent Savolainen

**Affiliations:** ^1^ BirdLife South Africa, Private Bag X16, Pinegowrie, Johannesburg 2123, South Africa; ^2^ Flyway Conservation Programme, Royal Society for the Protection of Birds, The Lodge, Sandy SG19 2DL, UK; ^3^ Department of Life and Consumer Sciences, School of Agriculture and Life Sciences, College of Agriculture and Environmental Sciences, University of South Africa, Private Bag X6, Pretoria, Florida 1710, South Africa; ^4^ Department of Plant and Environmental Biology, University of Ghana, P.O. Box LG 55, Legon, Acca, Ghana; ^5^ Université Nangui Abrogoua, Abidjan 02 BP 801, Côte d'Ivoire; ^6^ Kwame Nkrumah University of Science and Technology (KNUST), PMB, Kumasi, Ghana; ^7^ Laboratory of Applied Ecology, Faculty of Agricultural Sciences, University of Abomey-Calavi, Cotonou 01 BP 526, Benin; ^8^ UMR Agroécologie, Université de Bourgogne, INRAE 17 rue de Sully, Dijon 21000, France; ^9^ Bhejane Adventures, Wittedrift, Plettenberg Bay, Western Cape, South Africa; ^10^ Georgina Mace Centre for the Living Planet, Imperial College London, Silwood Park Campus, Ascot SL5 7PY, UK; ^11^ Centre National de recherche Agronomique, Marc Delorme Research Centre, Abidjan 07 BP 13, Côte d'Ivoire; ^12^ Department of Ecology and Evolutionary Biology, University of Tennessee, Knoxville, TN 37996, USA; ^13^ Department of Geography, Environmental Management and Energy Studies, University of Johannesburg, APK Campus, Auckland Park, Johannesburg 2006, South Africa; ^14^ Faculty of Agronomy, University of Parakou, Parakou BP 123, Benin; ^15^ School of Animal, Rural & Environmental Sciences, Nottingham Trent University, Nottingham, UK; ^16^ IUCN, Cambridge, UK; ^17^ Fauna & Flora, The David Attenborough Building, Pembroke Street, Cambridge, UK; ^18^ School of Management and Plant Seed Production, National University of Agriculture, P.O. 42 Box 43, Ketou, Benin; ^19^ Center of Excellence in Biodiversity and Natural Resource Management, University of Rwanda, Butare, Rwanda; ^20^ School for the Environment, University of Massachusetts-Boston, Boston, MA, USA; ^21^ Mabula Ground Hornbill Project, P.O. Box 876, Bela Bela 0480, South Africa; ^22^ Hornbill Specialist Group, IUCN Species Survival Commission, Rue Mauverney 28, 1196 Gland, Switzerland; ^23^ A Rocha Ghana, 4 Sabblah Link, Accra, Greater Accra Region, Ghana; ^24^ Nature Kenya, P.O. Box 44486, Nairobi, Kenya; ^25^ BirdLife International, Africa Partnership Secretariat, P.O. Box 3502—00100, Nairobi, Kenya; ^26^ Department of Biology, University of Oxford, Oxford OX1 3SZ, UK; ^27^ Agricultural Research Institute for Development—IRAD, P.O. Box 2123, Yaounde, Cameroon; ^28^ Laboratory of Applied Biology and Ecology, Faculty of Science, University of Dschang, P.O. Box 67, Dschang, West Region, Cameroon; ^29^ Department of Animal Biology and Conservation Science, University of Ghana, P.O. Box LG25, Legon, Accra, Ghana; ^30^ IUCN Species Survival Commission, Instituto Venezolano de Investigaciones Científicas (IVIC) and Provita, Caracas, Venezuela; ^31^ Applied Behavioural Ecological and Ecosystem Research Unit (ABEERU), University of South Africa, Private Bag X6, Pretoria, Florida 1717, South Africa; ^32^ Royal Botanic Gardens, Kew, Richmond TW9 3AB, UK

**Keywords:** Africa, key biodiversity areas, 30×30 target, indigenous peoples and local communities, green bonds, robotics and autonomous systems

## Abstract

Africa boasts high biodiversity while also being home to some of the largest and fastest-growing human populations. Although the current environmental footprint of Africa is low compared to other continents, the population of Africa is estimated at around 1.5 billion inhabitants, representing nearly 18% of the world’s total population. Consequently, Africa’s rich biodiversity is under threat, yet only 19% of the landscape and 17% of the seascape are under any form of protection. To effectively address this issue and align with the Convention on Biological Diversity’s ambitious ‘30 by 30’ goal, which seeks to protect 30% of the world’s land and oceans by 2030, substantial funding and conservation measures are urgently required. In response to this critical challenge, as scientists and conservationists working in Africa, we propose five recommendations for future directions aimed at enhancing biodiversity conservation for the betterment of African society: (i) accelerate data collection, data sharing and analytics for informed policy and decision-making; (ii) innovate education and capacity building for future generations; (iii) enhance and expand protected areas, ecological networks and foundational legal frameworks; (iv) unlock creative funding channels for cutting-edge conservation initiatives; and (v) integrate indigenous and local knowledge into forward-thinking conservation strategies. By implementing these recommendations, we believe Africa can make significant strides towards preserving its unique biodiversity, while fostering a healthier society, and contributing to global conservation efforts.

This article is part of the discussion meeting issue ‘Bending the curve towards nature recovery: building on Georgina Mace's legacy for a biodiverse future’.

## 1. Introduction

**Figure 1.**
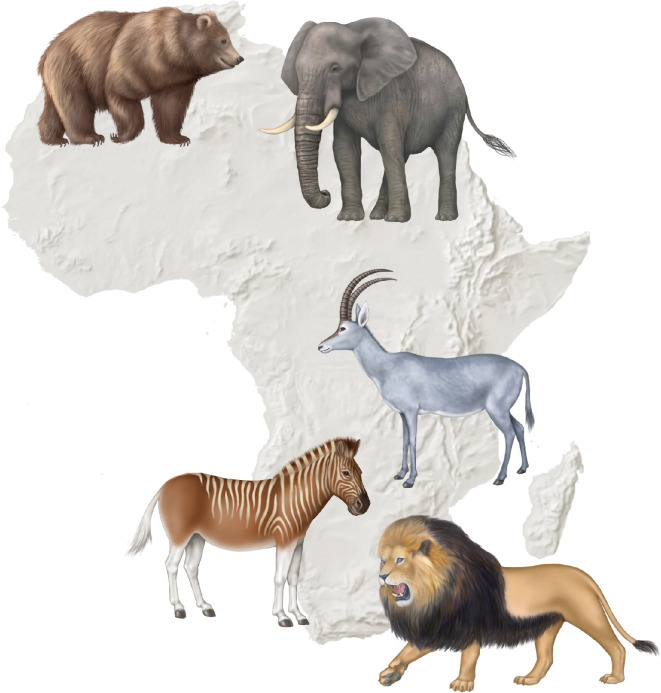
Africa has experienced the extinction of several taxa over the years due to over-hunting, among other causes. Charismatic examples include: (i) Atlas bear (*Ursus arctos crowtheri*), the only bear subspecies native to Africa, it lived in the Atlas Mountains of North Africa; (ii) North African elephant (*Loxodonta africana pharaohensis*), once inhabited North Africa and went extinct during Roman times, in part due to many being killed in circus games; (iii) blue antelope (*Hippotragus leucophaeus*), native to the southwestern coastal regions of South Africa, it became extinct in the late eighteenth century due to overhunting and habitat destruction; (iv) quagga (*Equus quagga quagga*), extinct in the wild by the late nineteenth century, with the last known individual dying in captivity in 1883; and (v) Cape lion (*Panthera leo melanochaita*), once inhabited the Cape of Good Hope and was hunted to extinction by the mid-nineteenth century. However, it is important to note that hunting, including trophy hunting, is not always detrimental to wildlife. In fact, it can sometimes benefit biodiversity conservation [3,4]. (Image credit: Debbie Maizels.)

Although biodiversity is key to ensuring humanity’s future [1], it is declining at unprecedented rates, with an estimated 1 million animal and plant species threatened with extinction [2]. Several charismatic species have been driven to extinction due to historic overhunting (see figure 1). To reverse biodiversity loss and highlight nature’s contributions to society, major international policy frameworks, such as the United Nations Sustainable Development Goals (SDGs), the Convention on Biological Diversity (CBD) and most recently the Kunming–Montreal Global Biodiversity Framework (KMGBF), have been developed. Under the CBD framework, a significant ambition was adopted at the 15th Conference of Parties (COP15) of the CBD in December 2022: to protect 30% of terrestrial, inland waters, coastal and marine areas by 2030. This aim focuses particularly on areas of high biodiversity importance, while ensuring they are effectively conserved and managed through systems of protected areas and other effective area-based conservation measures (OECMs). This ambition is collectively referred to as the UN ‘30 × 30’ target.

The African continent, constituting 20% of the world’s land, is pivotal in achieving this UN 30 × 30 target. Africa’s biodiversity is exceptional. The continent is home to over 70 000 plant species (about a sixth of all plant species globally), 1100 species of mammals (about 17% of the planet’s mammals), 2500 birds, 950 amphibians, about 2000 reptiles and 5000 freshwater fish species, not to mention the myriads of invertebrates [5]. Additionally, Africa is home to 8 of the 36 recognised global biodiversity hotspots [6], 373 wetlands of international importance (Ramsar sites), over 1255 Important Bird and Biodiversity Areas [7–9] and 1966 Key Biodiversity Areas (KBAs) [10]. Africa hosts 20% of the world’s rainforest areas, especially the Congo Basin, a 240-million-hectare rainforest spanning eight African countries and supporting the livelihoods of 100 million people in the region. The Congo Basin absorbs 4% of global carbon emissions annually, offsetting more than the continent’s annual emissions [9]. Africa’s biodiversity is unique, encompassing relatively intact Pleistocene fauna such as megaherbivores, both extant and extinct primate lineages ancestral to humans, the world’s highest extratropical plant and invertebrate biodiversity and endemism (e.g. in the Cape Floristic Region), and extraordinary radiations of species in Madagascar. Marine life is exceptional as well, with coral reefs stretching from Egypt to South Africa, providing millions of Africans with food. Coastal Atlantic and Pacific waters along Africa have impressive biological productivity and contribute 20% of the world’s fish harvest [9]. Despite this high biodiversity, many threats are having detrimental long-term impacts on both biodiversity and the ecosystem services it provides to humankind.

Given Africa’s rich biodiversity and the imminent challenges to address the many threats to biodiversity conservation, we present an African perspective on addressing the biodiversity crisis on the continent. BSB and VS contacted 49 conservationists and researchers in Africa, asking for their opinions on (i) the three key challenges hampering the achievement of biodiversity conservation for the betterment of African societies, and (ii) three key recommendations to effectively address these issues and align with the CBD’s ambitious 30 × 30 target. From the initial list of people contacted in October 2023, 25 responded, and their responses were broadly aggregated into five major recommendations, reflecting similar themes. These 25 respondents are the authors of this article, together with BSB and VS.

We recognize that the challenges and recommendations formulated here may not represent a purely endogenous African perspective. However, we acknowledge that collectively, we have a long experience delivering conservation outcomes in Africa, ranging from research, capacity building, conservation actions at species and landscape levels, to advocacy, policy and decision-making. We have collectively worked in more than 80% of African countries (44/54). Additionally, some of us have played important roles in convening African dialogues at major continental events like the African Protected Areas Congress (APAC), IUCN African Conservation Forums, Pan-African Ornithological Congress (PAOC), and representing an African voice at international policy discussion and negotiation forums like the IUCN Regional Conservation Forums and National Committees, CBD Conference of the Parties (COP), the Convention on the Conservation of Migratory Species of Wild Animals (CMS) and the United Nations Framework Convention on Climate Change (UNFCCC).

### (a) Threats to African biodiversity

On the African continent, only a small proportion of biodiversity has been assessed using globally recognized standards like the IUCN Red List of Threatened Species (https://www.iucnredlist.org/). For example, only around 37 000 African species have been evaluated using the IUCN Red List criteria [11]. Additionally, 4134 plants and 1901 animal species are listed under the Convention on International Trade in Endangered Species of Wild Fauna and Flora (CITES) as being threatened with extinction [12]. Some degree of protection is provided in 1812 African national parks, yet only 19% of Africa’s landscape and 17% of the seascape are under formal protection [13]. Although 52 out of 54 African countries are signatories of the CBD, biodiversity in Africa remains indeed severely threatened. What are the major causes? Key economic sectors contributing to biodiversity loss in Africa include agriculture, forestry, fishing, mining and urban expansion. Major drivers behind these impacts are population growth, governance challenges, infrastructure development, capacity constraints and increasing domestic and international demands. We briefly review each of these factors in turn.

Africa’s rapidly growing human population drives increased demand for land and natural resources, leading to further habitat destruction and fragmentation. Urban areas are expanding at high rates in Africa [14], sometimes at the expense of natural habitats. However, urban expansion is also often seen as a solution to environmental problems because higher population density in cities leaves more land available for conservation. Recent findings show that regions with high biodiversity and endemism are frequently among the poorest, with limited capacity to address biodiversity decline [15]. The human population in Africa is projected to quadruple by 2100 [16], with significant migration to urban centres. In 2000, African cities covered over 33 000 km² and are predicted to increase their land cover by 600% over the next 30 years, the highest rate of urban expansion in the world [17]. Although Africa’s environmental footprint is still low compared to regions like the US, Europe or China, this expansion may accelerate deforestation and agricultural encroachment into critical habitats for biodiversity. Unlike in South–Central America and Asia, African farmers often lack access to fertilizers, necessitating the cultivation of larger previously uncultivated areas or frequent shifting of agricultural land to achieve similar productivity levels [18]. For example, the Congo Basin rainforest, which spans 240 million hectares, lost 16 million hectares between 2000 and 2014, primarily due to small-scale agriculture [19,20]. Additionally, Africa’s energy needs, particularly for cooking, heavily rely on fuelwood and charcoal. In the Democratic Republic of Congo, for instance, fuelwood constitutes 95% of energy needs, equating to an estimated 70 million cubic meters of wood annually [21]. Consequently, deforestation in Africa, and its associated biodiversity losses, is predominantly driven by the expansion of smallholder and subsistence agriculture, as well as the extraction of fuelwood and charcoal for domestic use. Furthermore, 600 000 km² of forest (30% of Africa’s rainforests) are under logging concessions, while only 12% are protected [22].

Overexploitation of natural resources due to fishing, hunting and the harvesting of plants and animals are major threats (e.g. see figure 1). Overfishing, for example, has led to the depletion of fish stocks in West Africa, jeopardizing food security and livelihoods. Africa’s blue economy, comprising ports, fisheries, tourism and other coastal economic activities, is projected to grow to $405 billion by 2030 [9]. However, repeated mass coral bleaching events in East Africa [23] could result in a 30% contraction of this sector, leading to the loss of livelihoods for millions of African fishermen [9]. Furthermore, freshwater ecosystems in river basins with large urban populations lacking sufficient water are likely to experience flows inadequate to maintain ecological processes, which will likely impact freshwater fish populations [24].

Illegal wildlife trading has become the fourth biggest international organized crime [25]. It is estimated that between 1.6 and 4.6 million tons of bushmeat are extracted each year from Central Africa alone [26]. Africa’s rich wildlife, including elephants and rhinos, are particularly targeted by poachers due to the high international demand for ivory, horns and other animal parts. The illegal wildlife trade poses a severe threat to many species and undermines conservation efforts [9].

Climate change will affect Africa severely. Climate change exacerbates other threats by altering habitats and species distributions. Although it is still debatable what will happen in various parts of Africa, and whether it will become drier or wetter, projections indicate more extreme changes for rainforest regions with an estimated 3−4°C increase in temperature by 2100 [27,28]. This magnitude of temperature increase is thought to potentially destabilize the African tropical forest carbon sink, risk local extinction of more than 50% of plant, vertebrate and insect species across one-fifth of Africa, and risk total extinction of a third of freshwater fish and more than 90% of warm-water coral reefs [29]. Some of the most critically endangered reefs are in Madagascar, the Comoros and Mascarene Islands [9].

Pollution from agricultural runoff, pesticide use, artisanal mining, industrial activities and urbanization contributes to the degradation of freshwater ecosystems and harms wildlife. Soil erosion, salinisation and loss of soil fertility are widespread, affecting about 20% of Africa’s land surface [9]. This has serious implications for food security, threatening the achievement of SDGs 1, 2, 3 and 6 related to no poverty, zero hunger, good health and wellbeing, clean water and sanitation.

Non-native species, particularly those that become invasive and were introduced intentionally or accidentally, can outcompete, prey on or bring diseases to native species, leading to declines and sometimes extinction of native biodiversity. Invasive species disrupt ecosystem functions and can cause significant ecological and economic damages. Examples include the water hyacinth in Lake Victoria and the Nile perch, which have had significant impacts on aquatic ecosystems [30].

All the above-mentioned factors are likely to contribute to intensifying social problems, including mass population migrations and increased conflicts. Conflict in Africa will be more likely in the future because when populations are dense, young and growing rapidly, the conditions are primed for conflict [31]. In fact, globally, countries with higher population growth rates experience greater levels of social conflict. The age structure on the continent is relatively young, with 43% of its population being less than 15 years and 60% less than 24 years [32,33]. The World Bank estimates that by 2050 there will be 86 million internally displaced climate migrants in sub-Saharan Africa, with East Africa alone contributing to an estimated 10.1 million climate migrants [34].

Underlying drivers contributing to biodiversity loss in Africa include poor governance, limited technical capacity, inadequate infrastructure and resource grabs by foreign powers [9]. Particularly, ineffective governance, including in scientific institutions, and corruption severely hinder conservation efforts. Poorly enforced environmental regulations and lack of political will lead to illegal activities such as poaching and unregulated logging encroachment into protected areas, exacerbating biodiversity loss [9].

Many African institutions have insufficient technical expertise and resources to effectively document and manage biodiversity. Capacity-building initiatives, like those by the JRS Biodiversity Foundation, are essential to improve local expertise in biodiversity informatics and conservation strategies [35]. Limited access to remote conservation areas and inadequate facilities for scientific research impede effective biodiversity monitoring and protection efforts. This insufficiency in infrastructure can prevent timely responses to threats and reduce the overall effectiveness of conservation programmes [9]. Unfortunately, Africa’s rich native biodiversity remains poorly documented, mapped and protected [36].

Mining, logging and large-scale agricultural projects driven by foreign investments frequently disregard environmental sustainability [37], leading to significant biodiversity loss. The African continent, containing approximately 30% of the world’s minerals, is a hotspot for resource extraction. China’s investment in Africa is growing rapidly, with around $362 billion invested between 2005 and July 2019. Total trade between China and Africa increased from $9 billion in 2000 to $175 billion in 2015, making China Africa’s largest trade partner.

## 2. Recommendations

We propose five broad recommendations to address the biodiversity crisis and support sustainable development in Africa. Additionally, we provide African examples of where these recommendations are currently being implemented and show how they can benefit both the conservation of biodiversity and local people.

### (a) Accelerate data collection, data sharing and analytics for informed policy and decision-making

Our understanding of biodiversity risks in Africa, including major areas like the Congo Basin, remains limited. Resources are sparse, and information is fragmented. For instance, a global evaluation of reptile conservation status identified Central Africa as the region with the most significant data deficiencies [38]. There is an urgent need to collect, compile, share and analyse biodiversity data to inform policies and present this information in an accessible format for governments and non-scientists. More trained personnel, citizen scientists and community involvement are needed, but technological advances can also accelerate the gathering of biodiversity information [39].

Monitoring biodiversity is time-consuming and expensive to replicate spatially and temporally [40]. In the future, robotics and autonomous systems technologies combined with artificial intelligence (AI) could significantly facilitate data collection over large spatial and temporal scales by sensing, analysing and monitoring species’ populations across all taxa [41,42]. Recent technological advancements offer potential solutions for fully automated monitoring of ecological communities through increasingly affordable high-throughput recording hardware, which can collect rich multidimensional data [42]. Furthermore, incorporating machine learning into ecological workflows could improve inputs for models and lead to integrated tools for wildlife conservation [43].

There are already success stories in Africa that open the door to further developments. For example, 225 cameras were deployed in Serengeti National Park in Tanzania [44]. Analysing 1.3 million images, scientists were able to document the presence of 40 mammalian species in this savannah landscape [44]. In this case, AI was also able to validate large volumes of data submitted by citizen scientists. Similarly, in Algeria, infrared camera traps were deployed between 2008 and 2021 in the Bechar, Hoggar-Tassili and Ahaggar areas, which have enabled conservationists to document the presence of the Saharan cheetah (*Acinonyx jubtaus hecki*) and the Atlas leopard (*Panthera pardus panthera*), two subspecies believed to have gone extinct [45]. As another example, the K. Lisa Yang Center for Conservation Bioacoustics from Cornell University launched the Elephant Listening Project, in which acoustic monitoring is helping conserve forest elephants. Acoustic arrays can indeed help in tracking species movements, detecting poaching activities, rare and elusive species, leading to improved protection measures and a better understanding of species diversity, abundance, behaviour and population dynamics while contributing to better-informed conservation strategies and policies [44–47]. Additionally, with limited budget, which is often the case with conservation projects, the Conservation Area Prioritisation Through Artificial Intelligence (CAPTAIN) technique has been used to prioritize conservation areas, for example identifying areas with high numbers of endemic tree species threatened with extinction in Madagascar [39]. Therefore, increasingly in Africa and elsewhere in the world, AI techniques have proven important for the conservation of species and their critical habitats.

Despite these early successes, it is essential to ensure that these technological advances are accessible to conservationists, are culturally sensitive and involve local communities to achieve sustainable and effective results [48]. To enhance the efficiency of such techniques, the UK Robotics and Autonomous Systems Network for Environmental Sustainability rightly calls for the creation and funding of an integrated multidisciplinary task force and the development of education strategies to foster links between aspiring engineers, biologists and computer technologists. This could be incorporated into school curricula and continued into university and research facilities and is particularly relevant for Africa.

### (b) Innovate education and capacity building for future generations

Conservation efforts must be led by the communities who ultimately bear the costs and get the benefits. Although the African continent has a golden opportunity to develop innovative, home-grown solutions to its challenges [49], the training of conservation scholars and practitioners is an urgent task. Traditional mechanisms, such as funding research chairs at African universities, creating centres of research excellence and providing training within universities and national parks, as well as adequately funding African scholars to slow the brain drain, are essential [50]. A good example of such mechanisms includes the establishment of national funding schemes such as the National Research Foundation (NRF) in South Africa with a focus on funding research projects and excellent scientists through ‘NRF rating’, the latter allocated based on a researcher’s recent research outputs and impact as perceived by international peer reviewers. Most African universities have no such mechanisms, and this translates into a poor contribution of African researchers based in Africa to the global knowledge generation in the field of conservation. However, given the magnitude of the task, we must also explore innovative solutions to reach more students, researchers and conservationists in Africa. This includes finding ways of funding the scientists who are actively engaged in research and the practitioners who are directly enacting sound conservation strategies. Such funding mechanism needs to cut red tape and bring resources directly to scientists and practitioners. Additionally, African governments should be urged to reallocate their budget expenditure to include more funding to tertiary education and more opportunities for postgraduate studies. It is particularly important that postdoctoral fellowships are developed and funded by local government as a strategic choice to grow research productivity and boost innovation.

Notwithstanding, foreign countries can help. For example, since 1996, China’s involvement in training African university students has grown exponentially, and as of 2015, there were 50 000 African students in China. Therefore, China, among others, may play a crucial role in education and funding conservation on the continent in the future [37].

Here, we also propose establishing a hub for biodiversity conservation in Africa. This hub could be both distributed and virtual, serving as a central point for biodiversity data sharing, research, training and even community engagement, funding opportunities and collaboration (both North–South and, critically, South–South). It could function as a portal linking to other relevant biodiversity databases, whether global or local. Models for global hubs exist, such as GenBank for DNA sequence data and the Global Biodiversity Information Facility (GBIF) for biodiversity data. Establishing a focal point for African biodiversity resources would be highly beneficial. Given the multitude of existing initiatives in this area, young African researchers often find themselves overwhelmed and unsure of where to start. A centralized hub would streamline access to resources and enhance analytical capabilities, ultimately supporting and empowering the next generation of African researchers.

This hub could offer online training in R, the primary bioinformatics tool in biodiversity research today. It could also provide links to a comprehensive list of R packages for biodiversity analyses, including species accumulation curves, diversity indices, generalized linear models for analyzing species abundance and presence–absence, distance matrices and Mantel tests (e.g. CRAN, adiv, RBiodiversity). Training the new generation of taxonomists is also key to ensure continuity in conducting comprehensive biodiversity assessments. In addition, decolonizing curricula to include core courses in ecology and evolutionary biology can support effort to improve conservation science in Africa.

Additionally, the hub could align with localized and larger ongoing programmes, such as those by the African Academy of Sciences. It could also link to international initiatives like the capacity building and partnership programmes of the JRS Biodiversity Foundation, the Fulbright US Scholar Programme or the UK Commonwealth Awards, which offer opportunities for African scientists to teach, conduct research and carry out professional projects worldwide.

It is also important to ensure that trainees, especially those working in universities, are not overwhelmed with managerial duties at the expense of their research or conservation activities. Too often, young promising scientists are promoted to head of departments and consequently have no time for the biodiversity science that is critically needed in Africa.

For conservation initiatives to succeed, there must be a collective will from the population, including scientists, to advance conservation efforts. This can be challenging when other priorities, such as feeding one’s family or finding safe housing due to poverty and conflicts, rightfully take precedence.

Thus, education is crucial. We know that capacity-building programmes in developing countries and training local conservationists and communities significantly reduce habitat loss by enhancing the effectiveness of decision-making in conservation strategies and sustainable practices [51,52].

### (c) Enhance and expand protected areas, ecological networks and foundational legal frameworks

The effectiveness of Africa’s current network of protected and conserved areas in safeguarding biodiversity elements, including species, ecosystems and genetic diversity, can be questionable to a degree. Historically, many protected areas in Africa were not established or expanded with biodiversity conservation as the primary objective. Consequently, many threatened species, such as reptiles and amphibians, and their critical habitats are either absent from or situated outside the existing protected area networks [53–56].

To address these gaps, it is crucial to adopt unifying frameworks that identify areas of importance for biodiversity, covering different biodiversity elements and encompassing species across terrestrial, freshwater, marine and even subterranean realms. One effective approach is the identification of KBAs, which are sites that contribute significantly to the global persistence of biodiversity [57]. The KBA approach is science-based, harmonising multiple existing methods and applicable across all biogeographic realms [57,58]. Additionally, OECMs, which are areas that achieve long-term and effective *in situ* conservation of biodiversity outside protected areas, are vital [59]. Connecting these areas and creating wildlife corridors is also essential for supporting the migration and dispersal of species. Successful examples include the Kavango Zambezi Trans-frontier Conservation Area and the Zambezi-Chobe Floodplain Wildlife Dispersal Area [60,61].

To achieve Goal A and Target 3 of the CBD KMGBF, we advocate for increased political will for governments to adopt area-based conservation approaches, such as KBAs and OECMs. These approaches will directly inform where to expand protected and conserved areas in Africa. For example, in Mozambique, the identification of Mount Mabu forest as a KBA, and through targeted biodiversity surveys between 2008 and 2016, has led to the discovery of many endemic species that are threatened with extinction, such as the endangered Mount Mabu forest viper (*Atheris mabuensis*), near-threatened Mount Mabu chameleon (*Nadzikambia baylissi*), near-threatened Mount Mabu pygmy chameleon (*Rhampholeon maspictus*), endangered tropical mistletoe (*Helixanthera schizocalyx*) and endangered Mount Mabu horseshoe bat (*Rhinolophus mabuensis*) [62,63]. The KBA status of Mount Mabu forest and others has informed the expansion of the protected areas network in Mozambique, in which many species now benefit from better protection from damaging developments like mining and logging.

While several African countries have committed to the ambitious 30 × 30 goal, implementation bottlenecks remain a pressing concern with only 5 years left until 2030. To address these specific 30 × 30 challenges, it is noteworthy that recent actions by the Subsidiary Body on Implementation of the CBD targets, which met from 21 to 29 May 2024, in Nairobi, Kenya, have made significant progress. As a result of this meeting, five regional organizations in Africa were selected to bolster the implementation of the KMGBF: (i) the Central African Forest Commission (COMIFAC), (ii) the Ecological Monitoring Centre (CSE), (iii) the Regional Centre for Mapping of Resources for Development (RCMRD), (iv) the Sahara and Sahel Observatory (OSS) and (v) the South African National Biodiversity Institute (SANBI) [64]. However, the effectiveness of these organisations will depend on their technical and financial capacities.

To further ensure biodiversity conservation and societal sustainability, quantifiable metrics such as Species Threat Abatement and Restoration (STAR) are useful. These metrics help to identify the most important areas for mitigating different sorts of threats and inform habitat restoration initiatives [65]. Such initiatives should be integrated into various economic sectors that impact biodiversity to achieve both biodiversity and development outcomes. To assist African countries in mainstreaming biodiversity priorities into economic development activities, SANBI and the United Nations Environment Programme World Conservation Monitoring Centre (UNEP-WCMC) have produced a guideline document. These guidelines demonstrate how three headline indicators on the state of biodiversity and conservation spatial map products can focus interventions across landscapes and seascapes at the country level ([66,67] but see also [68]).

Given the anticipated significant growth of African cities, urban green spaces must also be designed to play a crucial role in conservation and climate change mitigation [69,70]. Urban green spaces serve a dual role of also connecting people with nature, especially in cities where that intimate relationship with nature no longer exists.

### (d) Unlock creative funding channels for cutting-edge conservation initiatives

Traditionally, conservation funding in Africa has predominantly come from outside Africa itself, for example through development assistance, philanthropists, foundations and trusts. Africa must now find its own funding routes to deliver long-lasting conservation benefits. A meta-analysis has revealed growing disparities in global conservation research capacity and its impact on biodiversity, with African countries being among the weakest [71]. Countries with the greatest need for biodiversity protection often lack the financial means to implement necessary changes. Africa exemplifies this paradox of wealth in biodiversity but economic hardship [15,72]. To address these challenges, we believe that Africa’s conservation sector must develop new, innovative, ‘bold’, but crucially long-term funding mechanisms to ensure biodiversity conservation [73].

An important component of this approach involves persuading both the private sector and African Union (AU) member states to invest in environmental protection. This is especially pressing given that 17 years ago, AU member states committed to spending 1% of their GDP on research and sustainable development, including conservation. However, their current spending averages only 0.42%, significantly below the global average of 1.7% [74]. It is widely acknowledged that traditional finance mechanisms alone cannot resolve Africa’s biodiversity crisis. Green finance can help (e.g. Rhino bonds), as well as conservation crowdfunding, payment for ecosystem services, biodiversity offsets, corporate conservation partnerships, carbon credits and conservation lotteries. These models can provide the financial sustainability needed for effective conservation.

However, it is essential to note that developing some of these innovative funding streams can take considerable time (typically between 3 and 30 years) depending on project size, type, location, participant structure or approval processes [75]. For instance, a partnership between government and civil society organizations in Sierra Leone (Conservation Society of Sierra Leone) and the United Kingdom (Royal Society for the Protection of Birds) transitioned a 20-year grant-funded project into a ‘Reducing Emissions from Deforestation and Forest Degradation in developing countries (REDD+)’ project 10 years ago. This Gola REDD + Project, registered under the Verra accounting standard, generates approximately 350 000 verified carbon units annually. Revenues from carbon sales helped protect the around 70 000 hectare Gola Rainforest National Park, benefiting over 120 local communities living at the forest edge. This partnership has therefore significantly contributed to social benefits and biodiversity conservation by restoring land degraded by commercial logging, agriculture and mining. Today, revenues from sales now employ 170 project staff and a benefit-sharing agreement with the seven Gola Chiefdoms focuses on these 120 forest-edge communities. As income from sales grows, revenue-sharing will be established with 50% to the Conservation Trust Fund (established via an Act of the Sierra Leone Parliament), 40% to provide sustainable incomes to Gola post-carbon project (ca. 2042) and 10% for civil society capacity building. Another compelling example of how long-term financing benefits both biodiversity conservation and local communities is seen in Shinyanga, Tanzania. Since 1985, carbon financing initiatives have facilitated the restoration of over 300 000 hectares of Miombo and *Acacia* woodlands. This large-scale ecological restoration has not only enhanced the resilience of local ecosystems but has also strengthened community resilience by promoting sustainable land-use practices [76].

Furthermore, in Benin’s Lama Forest Reserve, increased conservation funding significantly improved moist semi-deciduous forest cover by 99% from 2000 to 2022, reversing trends of habitat destruction and fragmentation [77,78]. Such positive land use—land cover change translates in reduced cover of the invasive weed *Chromolaena odorata* [79]. This also improves climate change mitigation with a 4.22-fold increase in annual carbon storage rate estimated at 13.95 tCO_2_eq year^-1^ ha^-1^ [78].

Finally, it is also important to recognize the potential for negative press and conflicts arising from some conservation projects. A notable example is the Bukaleba Forest Reserve in Uganda, where local communities were displaced, their livelihoods lost and minimal benefits reached them [80]. Therefore, managing stakeholder expectations is imperative, as well as being sensitive to Indigenous Peoples and Local Communities (IPLCs), as situations vary between countries and projects.

### (e) Integrate indigenous and local knowledge into forward-thinking conservation strategies

To secure a sustainable future for Africa, conservation stakeholders must actively engage IPLCs, ensuring their rights, cultures and sustainable practices are integral to future plans. IPLCs, who often reside in prime biodiversity areas and rely on sustainable use of biological resources, must be included through free, prior and informed consent (FPIC) to ensure long-term conservation success and resilience in Africa [1]. Co-creating the conservation agenda with IPLCs fosters inclusive discussions and establishes a roadmap for equitable governance. Additionally, biodiversity experts are often local people, exemplified by the work of African primatologists [81].

A review of over 160 articles demonstrates that IPLCs' central role in biodiversity conservation, especially when supported by legislation and policy, leads to fair and effective outcomes [82]. There is increasing evidence for greater convergence and complementarity between scientific research and local ecological knowledge [83–86]. Thus, incorporating conservation strategies inspired from local management schemes can foster increasing buy-in from local people but also fast track conservation actions. Although global and national acknowledgements, such as Goal C and Target 22 of the CBD KMGBF, highlight the importance of IPLC involvement, this is not consistently practiced in Africa. Local communities have often been displaced from their land for conservation reasons, creating a disincentive to engage [87], as seen in eastern, southern and western Africa, specifically Kenya, Mozambique and Ghana [88–90]. Consequently, many local communities do not support conservation interventions that require them to cede their land.

However, successful examples from Ghana illustrate that proper engagement and involvement of local communities in conservation governance can achieve a win–win outcome for both biodiversity and local people. The Community Resource Management Area (CREMA) initiative by the Ghana Wildlife Division of the Forestry Commission demonstrates this. CREMAs are geographic areas encompassing one or more local communities committed to sustainable natural resource management [89]. These community-based landscape governance systems strengthen multi-stakeholder collaboration, promoting biodiversity conservation and supporting wildlife migratory corridors. By diverting these corridors away from agricultural and development zones, they help balance ecological preservation with human land use [91].

The Murugu–Mognori CREMA exemplifies this approach. This CREMA was established through efforts starting in 2003 and officially inaugurated in 2008. It is located within the Busunu Traditional Area in northern Ghana and encompasses the Murugu and Mognori communities, which has emerged as a thriving model in the Mole landscape. It has significantly benefited the communities by supporting various livelihoods and fostering income-generating activities. For instance, a strategic business plan transformed the village into a vibrant eco-tourism destination, offering attractions such as canoe safaris, cultural performances and homestays. This initiative has created employment opportunities for 52 community members and is overseen by a 15-member board. Another pivotal achievement of this CREMA was the establishment of an organic shea butter processing centre, which has provided jobs for women from seven communities and enabled the export of processed shea products. Furthermore, a tree nursery has been established in Mognori community within this CREMA that nurtures up to a million indigenous seedlings, which are now used for habitat restoration but also generating employment for local women. These efforts culminated in the Sunkpa Shea Women Cooperative winning the prestigious Equator Prize Award in 2022 for their outstanding contribution to sustainable development. Thus, the Mole ecological landscape where this CREMA is situated offers a good model of mutual benefits for biodiversity and local communities, further exemplifying the vital contributions of IPLCs to conservation resilience. Over 500 CREMA members (172 males and 328 females) have now been trained in nursery management, organic farming, forest fire management, wildlife patrolling and ecological monitoring, leading to the restoration of over 800 hectares of degraded land. Through these activities, a wide range of species—including indigenous, economically important and conservation-priority plants such as shea (*Vitellaria paradoxa*), African locust beans (*Parkia biglobosa*), African mahogany (*Khaya senegalensis*) and rosewood (*Pterocarpus erinaceus*)—were extensively planted. This increased species diversity supports the proliferation of pollinators, which play crucial roles in maintaining vital ecosystem functions [92]. Additionally, four Conservation Agreements have been signed between CREMAs and The Savannah Fruits Company, where the community women group get additional bonuses for their organic shea butter production, amounting to over €28 700 annually.

Despite these successes, CREMAs encounter several challenges, including leadership transitions, the effects of rural–urban migration on the availability of skilled community members, and the need for careful beneficiary selection to sustain community support [90]. Overcoming these challenges highlights the crucial role of transparent management practices and strategic community engagement in maintaining the CREMA’s positive impact and advancing its goals of environmental conservation and economic empowerment.

Scaling up models such as CREMAs across Africa can incentivize local communities to conserve biodiversity while benefiting from their natural resources. Fortunately, similarand successful community-led initiatives already exist, such as the community conservancy model for wildlife protection in Kenya and Namibia [93,94] and biodiversity stewardship in South Africa [95].

## 3. Conclusion

Africa’s biodiversity showcases its unique natural wealth but faces mounting threats from rapid human population growth and unsustainable economic development. Protecting this biodiversity is crucial for all stakeholders, as it is intricately linked to the well-being of African society. Despite significant progress, major gaps in conservation efforts remain, especially in the face of climate change. However, numerous opportunities and initiatives can help address these knowledge and information gaps.

In the short term, setting up new protected areas, effectively managing existing protected areas, linking wildlife migratory corridors and networks, as well as improving education and increasing funding, should be prioritized to meet global targets such as the UN 30 × 30. In the longer term, robotics and artificial intelligence can aid conservation. Advancing equity, diversity and inclusion rooted in the Global South is also essential. Africa’s population is growing rapidly, and one effective way to address this growth is by focusing on the education and employment of youths and women. By linking these efforts to revenue-generating local conservation initiatives, we can create sustainable models that benefit both communities and the environment. Conservation practices must be led by local researchers and stakeholders, and international research must provide equitable workloads and recognition to African researchers [96].

Furthermore, communication and conservation optimism are key to ensuring effective messaging and reach out to the wider populations and decision-makers. To address this challenge, some early, yet commendable efforts have started with the support of the African Wildlife Foundation. At their recent meeting held in July 2024, they hosted the West African Journalism Workshop in Kinshasa, DRC. The journalists were trained to create uplifting and inspiring positive African conservation stories while recognising the role they can play in influencing public opinion to drive positive change.

Other country-specific or regional efforts, such as workshops on implementing the UN’s 30×30 protected areas target in South Africa, are admirable but need scaling up to inspire positive messaging on biodiversity and underscore the urgent need to conserve it for future generations.

The journey is long and will take time. However, with Africa’s abundance of ambitious, young, tech-savvy individuals, the future holds great promise.

## Data Availability

This article has no additional data.

## References

[1] Davies TJ , Maurin O , Yessoufou K , Daru BH , Bezeng BS , Schaefer H , Thuiller W , van der Bank M . Tree phylogenetic diversity supports nature’s contributions to people, but is at risk from human population growth. Ecology. (10.1101/2021.02.13.430985)

[2] Brondizio ES , Settele J , Díaz S , Ngo HT (eds). 2019 IPBES: global assessment report on biodiversity and ecosystem services of the Intergovernmental Science-Policy Platform on Biodiversity and Ecosystem Services. IPBES secretariat, Bonn, Germany. (10.5281/zenodo.3831673)

[3] Lindsey PA , Alexander R , Frank LG , Mathieson A , Romañach SS . 2006 Potential of trophy hunting to create incentives for wildlife conservation in Africa where alternative wildlife‐based land uses may not be viable. Anim. Conserv. **9** , 283–291. (10.1111/j.1469-1795.2006.00034.x)

[4] Di Minin E , Clements HS , Correia RA , Cortés-Capano G , Fink C , Haukka A , Hausmann A , Kulkarni R , Bradshaw CJA . 2021 Consequences of recreational hunting for biodiversity conservation and livelihoods. One Earth **4** , 238–253. (10.1016/j.oneear.2021.01.014)

[5] Chapman CA *et al* . 2022 The future of sub-Saharan Africa’s biodiversity in the face of climate and societal change. Front. Ecol. Evol. **10** , 790552. (10.3389/fevo.2022.790552)

[6] Archer E *et al* . 2018 Summary for policymakers of the regional assessment report on biodiversity and ecosystem services for Africa of the Intergovernmental Science-Policy Platform on Biodiversity and Ecosystem Services. Bonn, Germany: Intergovernmental Science-Policy Platform on Biodiversity and Ecosystem Services.

[7] Mittermeier RA , Turner WR , Larsen FW , Brooks TM , Gascon C . 2011 Global biodiversity conservation: the critical role of hotspots. In Biodiversity hotspots (eds FE Zachos , JC Habel ), pp. 3–22. Berlin, Germany: Springer. (10.1007/978-3-642-20992-5_1)

[8] Ajagbe AA . 2013 State of Africa’s birds: outlook for our changing environment. Nairobi, Kenya: BirdLife International Africa Partnership.

[9] Africa Center for Strategic Studies . 2022 Rising sea levels besieging Africa’s booming coastal cities. See https://africacenter.org/wp-content/uploads/2023/02/Rising-Sea-Levels-ENG.pdf.

[10] Key Biodiversity Areas . See https://www.keybiodiversityareas.org/ (accessed 2024).

[11] IUCN . 2024 *The IUCN red list of threatened species*. Version 2023-1. See https://www.iucnredlist.org.

[12] UNEP-WCMC . Species+. See https://www.speciesplus.net/ (accessed 2024).

[13] Discover the world’s protected and conserved areas. Protected Planet. See https://www.protectedplanet.net/en (accessed 2024).

[14] Güneralp B , Lwasa S , Masundire H , Parnell S , Seto KC . 2017 Urbanization in Africa: challenges and opportunities for conservation. Environ. Res. Lett. **13** , 015002. (10.1088/1748-9326/aa94fe)

[15] Rodríguez JP *et al* . 2022 Addressing the biodiversity paradox: mismatch between the co-occurrence of biological diversity and the human, financial and institutional resources to address its decline. Diversity **14** , 708. (10.3390/d14090708)

[16] UN . 2015 World population prospects: the 2015 revision, key findings and advance tables. Working paper ESA/P/WP.241. New York, NY: United Nations Department of Economic and Social Affairs.

[17] Seto KC , Fragkias M , Güneralp B , Reilly MK . 2011 A meta-analysis of global urban land expansion. PLoS One **6** , e23777. (10.1371/journal.pone.0023777) 21876770 PMC3158103

[18] Salerno J *et al* . 2018 Park isolation in anthropogenic landscapes: land change and livelihoods at park boundaries in the African Albertine Rift. Reg. Environ. Change **18** , 913–928. (10.1007/s10113-017-1250-1)

[19] Tyukavina A , Hansen MC , Potapov P , Parker D , Okpa C , Stehman SV , Kommareddy I , Turubanova S . 2018 Congo Basin forest loss dominated by increasing smallholder clearing. Sci. Adv. **4** , eaat2993. (10.1126/sciadv.aat2993) 30417092 PMC6221539

[20] Reiche J *et al* . 2021 Forest disturbance alerts for the Congo Basin using Sentinel-1. Environ. Res. Lett. **16** , 024005. (10.1088/1748-9326/abd0a8)

[21] Mayaux P *et al* . 2013 State and evolution of the African rainforests between 1990 and 2010. Phil. Trans. R. Soc. B **368** , 20120300. (10.1098/rstb.2012.0300) 23878331 PMC3720022

[22] Laporte NT , Stabach JA , Grosch R , Lin TS , Goetz SJ . 2007 Expansion of industrial logging in central Africa. Science **316** , 1451–1451. (10.1126/science.1141057) 17556578

[23] Hoegh-Guldberg O *et al* . 2007 Coral reefs under rapid climate change and ocean acidification. Science **318** , 1737–1742. (10.1126/science.1152509) 18079392

[24] McDonald RI , Green P , Balk D , Fekete BM , Revenga C , Todd M , Montgomery M . 2011 Urban growth, climate change, and freshwater availability. Proc. Natl Acad. Sci. USA **108** , 6312–6317. (10.1073/pnas.1011615108) 21444797 PMC3076880

[25] Wasser SK , Brown L , Mailand C , Mondol S , Clark W , Laurie C , Weir BS . 2015 Genetic assignment of large seizures of elephant ivory reveals Africa’s major poaching hotspots. Science **349** , 84–87. (10.1126/science.aaa2457) 26089357 PMC5535781

[26] Ingram DJ *et al* . 2021 Wild meat is still on the menu: progress in wild meat research, policy, and practice from 2002 to 2020. Annu. Rev. Environ. Resour. **46** , 221–254. (10.1146/annurev-environ-041020-063132)

[27] Zelazowski P , Malhi Y , Huntingford C , Sitch S , Fisher JB . 2011 Changes in the potential distribution of humid tropical forests on a warmer planet. Phil. Trans. R. Soc. B **369** , 137–160. (10.1098/rsta.2010.0238) 21115517

[28] Malhi Y , Adu-Bredu S , Asare RA , Lewis SL , Mayaux P . 2013 African rainforests: past, present and future. Phil. Trans. R. Soc. B **368** , 20120312. (10.1098/rstb.2012.0312) 23878339 PMC3720030

[29] Warren R , Price J , Graham E , Forstenhaeusler N , VanDerWal J . 2018 The projected effect on insects, vertebrates, and plants of limiting global warming to 1.5°C rather than 2°C. Science **360** , 791–795. (10.1126/science.aar3646) 29773751

[30] African Parks . 2024 African parks’ impact on achieving the UN sustainable development goals. See https://www.africanparks.org/sustainable-development-goals.

[31] Bradshaw CJA *et al* . 2021 Underestimating the challenges of avoiding a ghastly future. Front. Conserv. Sci. **1** , 615419. (10.3389/fcosc.2020.615419)

[32] He W , Goodkind D , Kowal PR . 2016 An aging world: 2015. Int. Popul. Rep. **95** , 1–165. (10.13140/RG.2.1.1088.9362)

[33] Juju D *et al* . 2020 Sustainability challenges in sub-Saharan Africa in the context of the sustainable development goals (SDGs). In Sustainability challenges in sub-Saharan Africa l: continental perspectives and insights from western and central Africa (eds A Gasparatos , M Ahmed , A Naidoo , K Karanja , O Fukushi ), pp. 3–50. Singapore, Singapore: Springer. (10.1007/978-981-15-4458-3_1)

[34] Rigaud KK et al . 2018. Groundswell: preparing for internal climate migration. Washington, DC: World Bank. (10.1596/29461).

[35] JRS Biodiversity Foundation . Capacity building and partnerships. See https://jrsbiodiversity.org/our-programs (accessed 2024).

[36] Achieng AO *et al* . 2023 Monitoring biodiversity loss in rapidly changing afrotropical ecosystems: an emerging imperative for governance and research. Phil. Trans. R. Soc. B **378** , 20220271. (10.1098/rstb.2022.0271) 37246384 PMC10225856

[37] Kalu K , Aniche ET . 2020 China–Africa economic relation: a double-edged sword for Africa. Afr. J. Econ. Sustain. Dev. **7** , 374. (10.1504/AJESD.2020.106827)

[38] Böhm M *et al* . 2013 The conservation status of the world’s reptiles. Biol. Conserv. **157** , 372–385. (10.1016/j.biocon.2012.07.015)

[39] Silvestro D , Goria S , Sterner T , Antonelli A . 2022 Improving biodiversity protection through artificial intelligence. Nat. Sustain. **5** , 415–424. (10.1038/s41893-022-00851-6) 35614933 PMC7612764

[40] Kallimanis AS , Mazaris AD , Tsakanikas D , Dimopoulos P , Pantis JD , Sgardelis SP . 2012 Efficient biodiversity monitoring: which taxonomic level to study? Ecol. Indic. **15** , 100–104. (10.1016/j.ecolind.2011.09.024)

[41] Corcoran E , Winsen M , Sudholz A , Hamilton G . 2021 Automated detection of wildlife using drones: synthesis, opportunities and constraints. Methods Ecol. Evol. **12** , 1103–1114. (10.1111/2041-210X.13581)

[42] Besson M , Alison J , Bjerge K , Gorochowski TE , Høye TT , Jucker T , Mann HMR , Clements CF . 2022 Towards the fully automated monitoring of ecological communities. Ecol. Lett. **25** , 2753–2775. (10.1111/ele.14123) 36264848 PMC9828790

[43] Tuia D *et al* . 2022 Perspectives in machine learning for wildlife conservation. Nat. Commun. **13** , 792. (10.1038/s41467-022-27980-y) 35140206 PMC8828720

[44] Swanson A , Kosmala M , Lintott C , Simpson R , Smith A , Packer C . 2015 Snapshot Serengeti: high-frequency annotated camera trap images of 40 mammalian species in an African savanna. Sci. Data **2** , 150026. (10.1038/sdata.2015.26) 26097743 PMC4460915

[45] Berthaud-Clair S . 2020 Dans le sud de l’Algérie, le guépard saharien, « dernier espoir » avant une disparition annoncée. Le Monde. 7 July 2020 See https://www.lemonde.fr/afrique/article/2020/07/07/dans-le-sud-de-l-algerie-le-guepard-saharien-dernier-espoir-avant-une-disparition-annoncee_6045410_3212.

[46] Wrege PH , Rowland ED , Keen S , Shiu Y . 2017 Acoustic monitoring for conservation in tropical forests: examples from forest elephants. Methods Ecol. Evol. **8** , 1292–1301. (10.1111/2041-210X.12730)

[47] Palmer MS , Swanson A , Kosmala M , Arnold T , Packer C . 2018 Evaluating relative abundance indices for terrestrial herbivores from large‐scale camera trap surveys. Afr. J. Ecol. **56** , 791–803. (10.1111/aje.12566)

[48] Wearn OR , Freeman R , Jacoby DMP . 2019 Responsible AI for conservation. Nat. Mach. Intell. **1** , 72–73. (10.1038/s42256-019-0022-7)

[49] Bizoza AR . 2024 How to meet Africa’s grand challenges with African know-how. Nature **629** , 10. (10.1038/d41586-024-01249-4) 38693405

[50] Schneider F , Patel Z , Paulavets K , Buser T , Kado J , Burkhart S . 2023 Fostering transdisciplinary research for sustainability in the Global South: pathways to impact for funding programmes. Hum. Soc. Sci. Commun. **10** , 620. (10.1057/s41599-023-02138-3)

[51] Brooks JS , Waylen KA , Borgerhoff Mulder M . 2012 How national context, project design, and local community characteristics influence success in community-based conservation projects. Proc. Natl Acad. Sci. USA **109** , 21265–21270. (10.1073/pnas.1207141110) 23236173 PMC3535631

[52] Cinner JE *et al* . 2012 Comanagement of coral reef social–ecological systems. Proc. Natl Acad. Sci. USA **109** , 5219–5222. (10.1073/pnas.1121215109) 22431631 PMC3325732

[53] Beresford AE , Buchanan GM , Donald PF , Butchart SHM , Fishpool LDC , Rondinini C . 2011 Poor overlap between the distribution of protected areas and globally threatened birds in Africa. Anim. Conserv. **14** , 99–107. (10.1111/j.1469-1795.2010.00398.x)

[54] Hoveka LN , van der Bank M , Bezeng BS , Davies TJ . 2020 Identifying biodiversity knowledge gaps for conserving South Africa’s endemic flora. Biodivers. Conserv. **29** , 2803–2819. (10.1007/s10531-020-01998-4)

[55] Oliveira-Dalland LG , Alencar LRV , Tambosi LR , Carrasco PA , Rautsaw RM , Sigala-Rodriguez J , Scrocchi G , Martins M . 2022 Conservation gaps for neotropical vipers: mismatches between protected areas, species richness and evolutionary distinctiveness. Biol. Conserv. **275** , 109750. (10.1016/j.biocon.2022.109750)

[56] Bakarr MI . 2023 Reimagining protected and conserved areas in Africa: perspectives from the first Africa Protected Areas Congress. Conserv. Lett. **16** , e12944. (10.1111/conl.12944)

[57] ICUN . 2016 A global standard for the identification of key biodiversity areas, 1st edn. Gland, Switzerland: IUCN.

[58] Plumptre AJ *et al* . 2024 Targeting site conservation to increase the effectiveness of new global biodiversity targets. One Earth **7** , 11–17. (10.1016/j.oneear.2023.12.007)

[59] WWF and IUCN WCPA . 2023 30 x 30. a guide to inclusive, equitable and effective implementation of target 3 of the kunming-montreal global biodiversity framework: version 1. Gland, Switzerland: WWF and IUCN World Commission on Protected Areas. See https://iucn.org/sites/default/files/2023-09/30x30-target-framework.pdf.

[60] Naidoo R , Kilian JW , Du Preez P , Beytell P , Aschenborn O , Taylor RD , Stuart-Hill G . 2018 Evaluating the effectiveness of local- and regional-scale wildlife corridors using quantitative metrics of functional connectivity. Biol. Conserv. **217** , 96–103. (10.1016/j.biocon.2017.10.037)

[61] Stoldt M , Göttert T , Mann C , Zeller U . 2020 Transfrontier conservation areas and human–wildlife conflict: the case of the Namibian component of the Kavango-Zambezi (KaZa) TFCA. Sci. Rep. **10** , 7964. (10.1038/s41598-020-64537-9) 32409783 PMC7224369

[62] Fisher J . 2024 Secret ‘sky island’ rainforest saved by new discoveries. BBC. See https://www.bbc.co.uk/news/articles/c51ylgr1zpxo.

[63] Key Biodiversity Areas Partnership . 2024 Key biodiversity areas factsheet: Mount Mabu (extracted from the World Database of Key Biodiversity Areas). See https://keybiodiversityareas.org/.

[64] Convention on Biological Diversity (CBD) . How the freshly selected regional centres will bolster the implementation of the KMGBF. See https://www.cbd.int/article/sbi4-regional-centres-implementation-2024 (accessed 9 June 2024).

[65] Mair L *et al* . 2021 A metric for spatially explicit contributions to science-based species targets. Nat. Ecol. Evol. **5** , 836–844. (10.1038/s41559-021-01432-0) 33833421

[66] SANBI and UNEP-WCMC . 2016 Mapping biodiversity priorities: a practical, science-based approach to national biodiversity assessment and prioritisation to inform strategy and action planning. Cambridge, UK: UNEP-WCMC.

[67] SANBI and UNEP-WCMC . 2024 Mapping biodiversity priorities: a practical approach to spatial biodiversity assessment and prioritisation to inform national policy, planning, decisions and action, 2nd edn. Pretoria, South Africa: South African National Biodiversity Institute.

[68] Vergez A . 2022 Mainstreaming biodiversity into priority economic sectors. Lessons from the assessment of main threats in 16 BIODEV2030 pilot countries. Gland, Switzerland: IUCN. (10.2305/IUCN.CH.2022.12.en)

[69] Ofori BY , Garshong RA , Gbogbo F , Owusu EH , Attuquayefio DK . 2018 Urban green area provides refuge for native small mammal biodiversity in a rapidly expanding city in Ghana. Environ. Monit. Assess. **190** , 480. (10.1007/s10661-018-6858-1) 30032389

[70] Anderson B , Patino Quinchia JE , Prieto Curiel R . 2022 Boosting African cities’ resilience to climate change: the role of green spaces. Paris, France: West African Papers, OECD Publishing. (10.1787/3303cfb3-en)

[71] Zhang L , Yang L , Chapman CA , Peres CA , Lee TM , Fan PF . 2023 Growing disparity in global conservation research capacity and its impact on biodiversity conservation. One Earth **6** , 147–157. (10.1016/j.oneear.2023.01.003)

[72] White & Case . 2023 Preserving Africa’s biodiversity: Why global funding is vital. See https://www.whitecase.com/insight-our-thinking/africa-focus-summer-2023-preserving-africas-biodiversity.

[73] Cabrera NH , Planitzer C , Yudelman T . 2021 Securing sustainable financing for conservation areas: a guide to project finance for permanence. Washington, DC: WWF.

[74] Caelers D , Okoth D . 2023 Research funding in africa: navigating sustainability and shifting perspectives. Nature Africa (10.1038/d44148-023-00360-4)

[75] Von Avenarius A , Devaraja TS , Kiesel R . 2018 An empirical comparison of carbon credit projects under the clean development mechanism and verified carbon standard. Climate **6** , 49. (10.3390/cli6020049)

[76] Barrow E . 2014 300,000 hectares restored in Shinyanga, Tanzania—but what did it really take to achieve this restoration. SAP. **7** . http://journals.openedition.org/sapiens/1542

[77] Padonou CSJ . 2021 Tracking and assessing the socio-economic impacts of conservation funding in benin over the long-term. Doctoral dissertation, University of Illinois at Urbana-Champaign.

[78] Biah I , Azihou AF , Guendehou S , Sinsin B . 2024 Land use/land cover change and carbon footprint in tropical ecosystems in Benin, West Africa. Trees For. People **15** , 100488. (10.1016/j.tfp.2023.100488)

[79] Sinasson GKS , Shackleton CM , Glèlè Kakaï RL , Sinsin B . 2017 Forest degradation and invasive species synergistically impact Mimusops andongensis (Sapotaceae) in Lama Forest Reserve, Benin. Biotropica **49** , 160–169. (10.1111/btp.12370)

[80] Schmid DV . 2023 Are forest carbon projects in Africa green but mean?: a mixed-method analysis. Clim. Dev. **15** , 45–59. (10.1080/17565529.2022.2054400)

[81] Hobaiter C , Akankwasa JW , Muhumuza G , Uwimbabazi M , Koné I . 2021 The importance of local specialists in science: where are the local researchers in primatology? Curr. Biol. **31** , R1367–R1369. (10.1016/j.cub.2021.09.034) 34699794

[82] Dawson NM *et al* . 2021 The role of indigenous peoples and local communities in effective and equitable conservation. Ecol. Soc. **26** , 19. (10.5751/ES-12625-260319)

[83] Gagnon CA , Berteaux D . 2009 Integrating traditional ecological knowledge and ecological science: a question of scale. Ecol. Soc. **14** , 1. (10.5751/ES-02923-140219)

[84] Tengö M , Brondizio ES , Elmqvist T , Malmer P , Spierenburg M . 2014 Connecting diverse knowledge systems for enhanced ecosystem governance: the multiple evidence base approach. Ambio **43** , 579–591. (10.1007/s13280-014-0501-3) 24659474 PMC4132468

[85] Braga‐Pereira F *et al* . 2022 Congruence of local ecological knowledge (LEK)‐based methods and line‐transect surveys in estimating wildlife abundance in tropical forests. Methods Ecol. Evol. **13** , 743–756. (10.1111/2041-210X.13773)

[86] Kor L , Fernández-Lucero M , Granados Flórez DA , Dawson TP , Diazgranados M . 2024 Bridging local and scientific knowledge for area-based conservation of useful plants in Colombia. Ambio **53** , 309–323. (10.1007/s13280-023-01921-5) 37828254 PMC10774498

[87] Lunstrum E . 2016 Green grabs, land grabs and the spatiality of displacement: eviction from Mozambique’s Limpopo National Park. Area **48** , 142–152. (10.1111/area.12121)

[88] Chabeda-Barthe J , Haller T . 2018 Resilience of traditional livelihood approaches despite forest grabbing: Ogiek to the west of Mau Forest, Uasin Gishu County. Land **7** , 140. (10.3390/land7040140)

[89] Arthur BC . 2000 Forestry Commission, Wildlife Division policy for collaborative community based wildlife management, September 2000. See https://newsite.fcghana.org/wildlife-issues/wildlife-division-policy-on-collaborative-community-based-wildlife-management-september-2000/.

[90] Logah V *et al* . 2024 Soil carbon, nutrient, and vegetation dynamics of an old Anogeissus grove in Mole National Park, Ghana. Biotropica **56** , e13299. (10.1111/btp.13299)

[91] Bayala ERC , Ros-Tonen M , Yanou MP , Djoudi H , Reed J , Sunderland T . 2024 Towards more inclusive community landscape governance: drivers and assessment indicators in Northern Ghana. For. Pol. Econ. **159** , 103138. (10.1016/j.forpol.2023.103138)

[92] Ollerton J . 2017 Pollinator diversity: distribution, ecological function, and conservation. Annu. Rev. Ecol. Evol. Syst. **48** , 353–376. (10.1146/annurev-ecolsys-110316-022919)

[93] Otianga-Owiti E , Okori JJL , Nyamasyo S , Amwata DA . 2021 Governance and challenges of wildlife conservation and management in Kenya. In Wildlife biodiversity conservation (eds SC Underkoffler , HR Adams ). Cham, Switzerland: Springer.

[94] Wenborn M , Svensson MS , Katupa S , Collinson R , Nijman V . 2022 Lessons on the community conservancy model for wildlife protection in Namibia. J. Environ. Dev. **31** , 375–394. (10.1177/10704965221121026)

[95] Rawat YS . 2017 Sustainable biodiversity stewardship and inclusive development in South Africa: a novel package for a sustainable future. Curr. Opin. Environ. Sustain. **24** , 89–95. (10.1016/j.cosust.2017.03.003)

[96] Ocampo-Ariza C *et al* . 2023 Global South leadership towards inclusive tropical ecology and conservation. Perspect. Ecol. Conserv. **21** , 17–24. (10.1016/j.pecon.2023.01.002)

